# No Simple Fluke: Schistosomiasis as an Emerging Regulator of Haematopoiesis

**DOI:** 10.1111/pim.70094

**Published:** 2026-07-16

**Authors:** Shinjini Chakraborty

**Affiliations:** ^1^ Division of Cancer Sciences, Faculty of Biology, Medicine and Health The University of Manchester Manchester UK

**Keywords:** cytokines, haematopoiesis, haematopoietic niche, schistosomiasis

## Abstract

Schistosomiasis, or bilharzia, is a highly prevalent water‐borne helminth infection that accounts for an estimated 1.5–1.7 billion disability‐adjusted life years lost annually. Pathology driven by adult worms and eggs is accompanied by robust immunomodulation in the human host. Protective responses may promote worm elimination, limit bystander tissue injury caused by parasite migration and contain eggs within granulomatous lesions. However, evidence also suggests that schistosomes can actively or indirectly influence haematopoiesis, thereby reshaping downstream mature immune cell responses. This perspective reviews current literature on schistosome‐dependent and host‐derived regulation of haematopoiesis, the importance of preclinical modelling and the emerging relevance of controlled human challenge models. It also discusses bone marrow organoid models and their utility for recapitulating complex infection dynamics, with the aim of informing clinically relevant studies and therapeutic strategies.

## 1 Introduction

Neglected tropical diseases (NTDs) constitute a substantial proportion of chronic macroparasitism and account for approximately 1.7 billion cases worldwide [1]. Among NTDs, schistosomiasis and its causative species continue to affect > 150 million people annually, with the highest disease burden carried by parts of Asia, Africa and South America [2]. Poor hygiene and sanitation often complicate schistosomiasis treatment, despite active mass‐drug administration programmes with praziquantel aimed at prophylactically treating patients from pre‐school age [3]. However, dependence on a single drug carries the risk of resistance and reduced efficacy in endemic regions until suitable alternatives or vaccines are available [3, 4].

**FIGURE 1:**
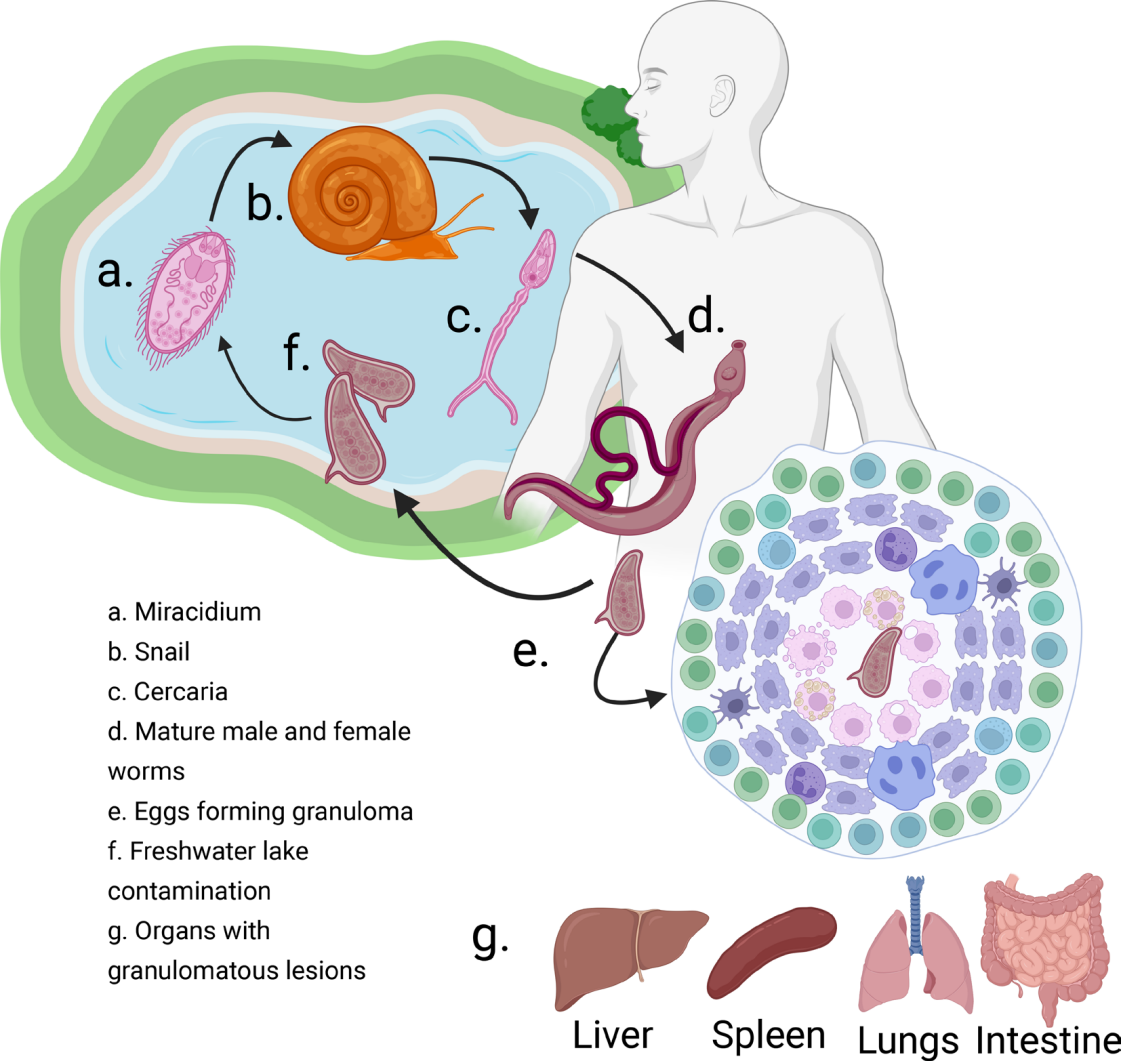
Life cycle of schistosomes and impact on its primary host. Schistosomal free‐living larval forms, cercariae, infect its primary human host via skin penetration. Worms undergo sexual maturation and start laying eggs after reaching patency. Eggs may either contaminate freshwater bodies through human excreta, where they hatch into a miracidium—the intermediate stage infecting snails. Cercariae developing within snails become sources of reinfection in endemic regions. Schistosome eggs can enter the circulation and transport to highly vascularised tissues, e.g., liver, lungs and spleen, recruit mature immune cells in large numbers, and lead to formation of fibrotic granulomatous lesions. Image generated using Biorender.

The schistosome life cycle alternates between freshwater snails and humans as the primary host [5]. Unhatched eggs from human excrement can contaminate freshwater (Figure 1f), hatch into a miracidium to infect snails (Figure 1a) and undergo asexual maturation to develop into its free‐swimming larval form or cercaria (Figure 1b) [5]. Humans get infected when in contact with contaminated waterbodies (Figure 1c). Schistosome cercariae invade the skin barrier, generating a highly localised inflammation at the initial or pre‐patency stage [6, 7]. Mature male and female worms start producing eggs after reaching patency, which, when released in large numbers, can migrate through the host vasculature, triggering localised inflammatory and fibrotic granulomas (Figure 1d,e) [8]. A high burden of granulomatous lesions results in an enlarged spleen and liver (hepato‐splenomegaly), thus exacerbating bystander tissue damage in patients at advanced stages of infection.

Pre‐empting any debilitating response is impossible by simply measuring egg burden (Kato‐Katz method), or by circulating levels of cathodic or anodic worm antigens [9, 10, 11]. Transitioning from a highly localised percutaneous inflammation within days, to a broadly regulatory/type 2 immune response in chronic infection involves several sub‐stages of acute inflammatory symptoms, e.g., Katayama fever [12, 13]. Alongside these host inflammatory sequelae, host cells must continuously respond to a net impact of schistosome‐dependent antigenic responses. More than 200 antigens expressed by schistosome cercariae, adult worms and eggs generate a formidable arsenal of immunomodulatory proteins [11]. Cercarial proteases and excretory/secretory (E/S) products, adult schistosomal worm proteins and soluble egg antigens (SEA) are known immunoregulatory mediators; their multitude of regulatory responses have been elaborately covered in reviews elsewhere [14, 15].

Schistosome‐infected patients often present with symptomatic anaemia, presently thought to result from worm‐migration‐associated tissue damage (internal bleeding, haematuria) [16, 17]. Causal mechanisms of this symptom are not fully understood at present. Additionally, anaemia can put demand‐adapted pressures on haematopoietic and haemostatic processes, drive aberrant erythropoietic output and contribute to overall poor patient‐health outcomes [18, 19].

**FIGURE 2:**
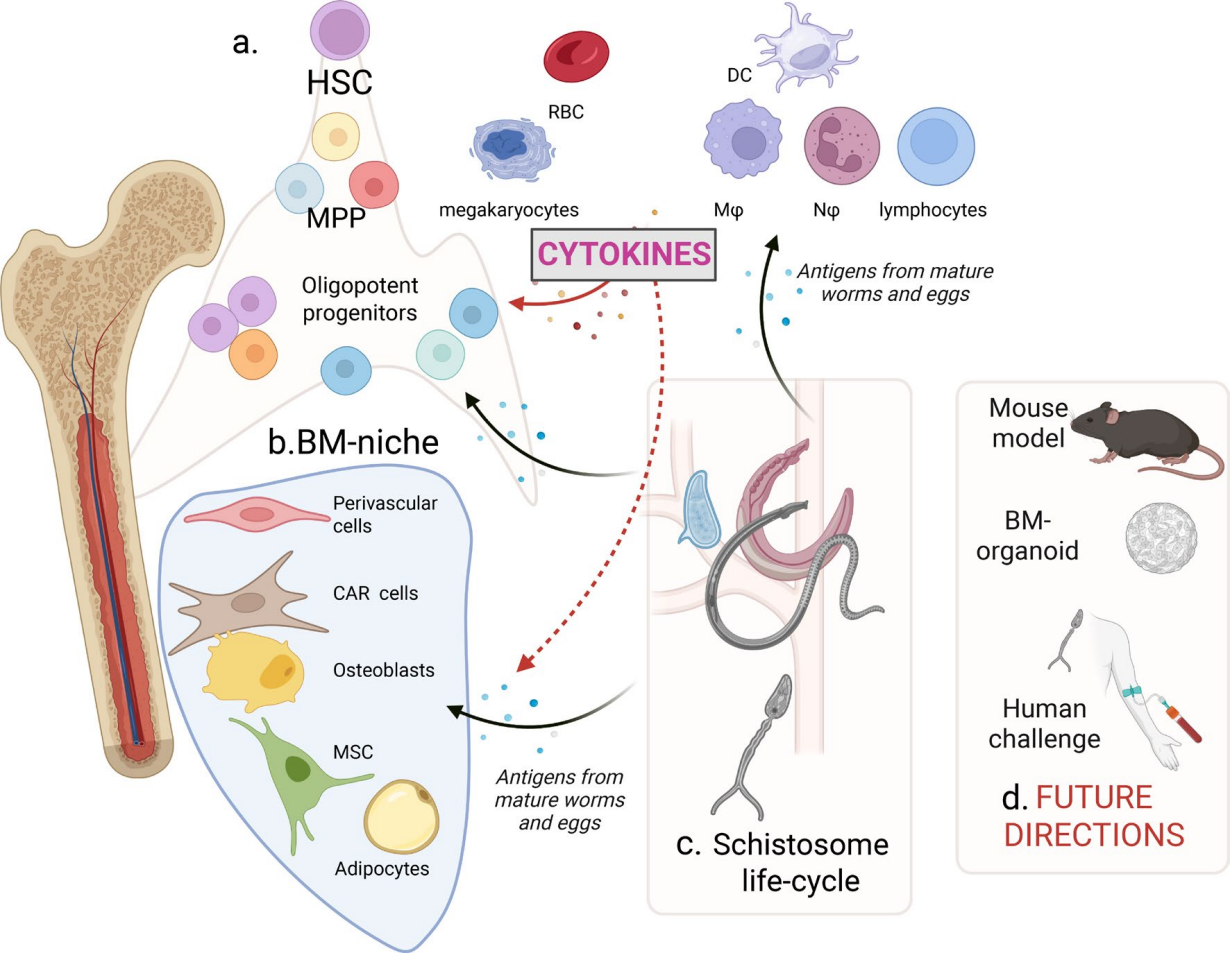
Haematopoiesis at the centre of schistosomiasis‐driven pathology. (a) Haematopoietic stem cells (HSC) are multipotent cells which can reconstitute all mature immune and non‐immune blood cell populations, such as—myeloid and lymphoid cells, red blood cells (RBC) and megakaryocytes and platelets. Intermediate lineage‐biased multipotent (MPP) and oligopotent progenitors with limited lineage potential feed into the haematopoietic cascade and generate mature blood cells. Bone marrow (BM) is the central organ of haematopoiesis. (b) Besides HSCs and other progenitors, BM‐niche cells are integral in supporting the haematopoietic cascade. Cytokines generated by activated immune cells can affect both, HSPCs and niche‐cells during infection and inflammation. (c) Schistosomal antigens generated in various life stages may influence both—HSPCs and niche‐cells, in inducing demand‐adapted haematopoiesis. (d) Future studies can include in vitro, in vivo and clinical controlled challenge models to emulate schistosomiasis‐dependent pathologies and study how haematopoiesis is altered in acute and chronic stages of infection. Image generated using Biorender.

The BM is a highly complex tissue—including haematopoietic stem and progenitor cells (HSPC), immune cells at various stages of maturation, stromal cell populations, osteoblasts, sinusoidal perivascular endothelial cells and adipocytes [20, 21]. Haematopoietic stem cells (HSCs) can generate all mature blood cell populations (red and white blood cells, megakaryocytes and platelets) and generally exist in BM sinusoids as a quiescent pool of cells (Figure 2a) [22]. They are characterised by their ability to intermittently divide for self‐renewal or differentiate into lineage‐restricted progenitors [20, 22]. Multipotent (MPPs) and oligopotent progenitors progressively acquire lineage restriction, owing to physiological and/or systemic cues (such as infection, injury and chronic inflammation) and replenish the reserves of all mature immune cell populations [23]. HSC quiescence and/or differentiation is actively supported by BM‐niche‐associated cells, which express a range of cytokines, chemokines and surface receptors essential for HSPC‐retention (Figure 2b) [21]. However, this niche organisation can be disturbed during stress haematopoiesis [24]. Lack of homing signals in the BM promotes HSPC egress, a common phenomenon seen during stress haematopoiesis and allows these cells to seed in distant extramedullary sites such as spleen [25, 26], liver [27], gingiva [28], lungs [29] and adrenal glands [30]. By contrast, schistosomiasis is a complex cytokine syndrome of type 1, type 2 and regulatory responses. Interferons (IFN), interleukin‐(IL) 4, IL‐5 and IL‐13 (type 2 response), transforming growth factor (TGF) β and IL‐10, IL‐17 and IL‐23 elevate in the host bloodstream, alluding to a multifarious effect on haematopoiesis and its downstream lineage maturation programmes [31, 32, 33, 34, 35].

As an emerging area of research, with only three preclinical studies to date [36, 37, 38]—there remains much to be explored into potential haematopoietic alterations during schistosomiasis. Moving beyond popularly studied type 1 immunity‐driven alterations, this review will summarise current knowledge of schistosome‐mediated haematopoietic regulation and discuss how preclinical mouse models, controlled human challenge studies and ex vivo 3D bone marrow organoid systems can be integrated to dissect schistosome‐dependent and host‐mediated mechanisms.

### 1.1 Preclinical Models of Schistosomiasis in Evaluating Host Haematopoietic Regulation

Cell‐intrinsic transcriptomic and epigenetic alterations, along with niche‐dependent extrinsic regulators, drive HSC exhaustion, differentiation and lineage commitment [22, 39, 40, 41, 42, 43, 44]. Although our current knowledge is primarily limited to bacterial, viral or fungal ligands that generate type 1 immune responses, pro‐inflammatory responses during severe schistosomiasis may have a similar impact on HSPCs [23, 45, 46, 47]. Murine schistosomiasis models can help bridge this knowledge gap initially. Preclinical studies may involve challenging mice with high numbers of cercaria (> 80 per mouse) to mimic short‐term acute infection [48]. As schistosomes reach patency within 6–7 weeks, mice develop granulomatous lesions in the liver and spleen. While worm burden determines the severity of these lesions, adult worms can cause significant tissue damage, thereby eliciting a complex host‐damage response. Using a high‐dose acute infection with ~200 cercariae, Wijshake et al. found monocytosis and eosinophilia in infected mouse BM, together with an overall decrease in central reserves of MPPs [36]. Along with erythropoietic progenitors, these cells seemed to have shifted to extramedullary sites [36]. When transplanted into BM‐depleted naïve recipients, the whole blood reconstitution potential of infected‐mouse HSPCs was found to be severely impaired [36].

By contrast, challenging mice with a lower 35–40 cercariae can be used to mimic chronic infection approximating patient‐relevant disease development [37, 38]. In a recent study modelling chronic schistosomiasis, Greenman et al. discovered that macrophage‐dependent platelet clearance drove infection‐associated thrombocytopenia, promoted the retention of immature megakaryocytes in BM, yet left megakaryocytic or erythroid progenitors largely unaffected [37]. As type 2 cytokines such as IL‐4, IL‐5 and IL‐13 predominate in chronic schistosomiasis, naïve or infected‐mouse HSCs were treated with IL‐4 in vitro to test their responses in differentiating conditions [37]. This experiment revealed that HSC colonies survived poorly and favoured myeloid bias at the expense of megakaryocytic differentiation (unpublished data). Together with these findings, it is reasonable to assume that schistosome‐driven haematopoietic responses cannot be solely attributed to type 2 cytokine‐driven responses. Further in vitro as well as in vivo experiments using combined cytokine challenges may help dissect how schistosome‐specific molecular programmes and resultant functional changes influence haematopoietic responses.

The impact of tissue‐damage during schistosome development and migration could be an integral driver of haematopoiesis as well. Alarmins released during host tissue damage, such as— ‐25 [49], thymic stromal lymphopoietin [50] and IL‐33 [51], which mobilise eosinophils, basophils, mast cells and macrophages to promote type 2 cytokine responses and fibrotic mechanisms for injury resolution [52]. Using a murine model of an intestinal helminth infection, another recent study showed that IL‐33 induces a basophilic–eosinophilic myelopoietic (BEM) bias in megakaryocytic/erythroid progenitors (MEPs) through transcriptional regulation [53]. A similar MEP‐to‐BEM lineage switch may be pertinent during the maturation phase of schistosomes; however, BM accumulation of related alarmins has not been shown in preclinical models so far.

Cell‐autonomous HSPC responses may also be supplemented/antagonised by changes within the haematopoietic niche, as has been shown in viral or bacterial in vivo models before. For example, exogenous cyclic diguanylate (ci‐Di‐GMP), a common signalling messenger in bacteria, can directly interact with BM‐niche mesenchymal stem cells (MSC) to reduce the expression of HSPC‐homing cytokines and surface receptors [54]. Wijshake et al. have already reported how acute schistosomiasis shifts haematopoiesis from central to peripheral tissues [36], but a schistosome infection‐dependent niche‐reconstruction during schistosomiasis still requires further elucidation.

HSPCs and niche cells isolated from pre‐clinical mouse models can inform us about enriched signalling cascades and their downstream impact on HSPC lineage‐commitment and function. Questions concerning how long these molecular changes are sustained in HSPCs can be addressed as praziquantel is equally effective in clearing worms from mice. Cured‐mouse HSPCs can be analysed for sustained chromatin/epigenetic alterations and the consequence of persisting egg antigens in driving these genomic changes. Evidence generated in preclinical models would eventually have to be translated in clinical settings. To that end, recent developments in conducting schistosome challenge studies have already set a promising precedent to phenotype patient‐HSPCs during schistosomiasis.

### 1.2 A Controlled Human Schistosomiasis Model

Clinical studies have majorly focused on correlating disease severity with serum cytokines' levels and circulatory worm/egg antigens and underscoring significant heterogeneity in infected‐patient prognosis. Acute schistosomiasis syndrome is presented with elevated levels of serum TNFα, IFNƔ, SEA‐specific IgE and circulatory immune‐complexes [55] and Th17 cytokines [31]. By contrast, immunologically silent stages of chronic schistosomiasis are characterised by high levels of serum IL‐4, IL‐5 and IL‐10 [56] and TGFβ [31]. However, pre‐defining patient‐responses solely depending on cytokine readouts may be inaccurate. Patients may develop severe hepatic pathology and portal hypertension at chronic stages of infection, then characterised by elevated levels of serum IL‐1β, IL‐2, IL‐18, GM‐CSF, TNF (type 1) and IL‐9, IL‐17 and IL‐23 (Th17) cytokines [31, 33, 57] triggering a sequela of local and systemic responses. So far, such studies have broadly depended on patients presenting late‐schistosomiasis‐related pathologies only.

The latest development of controlled schistosomiasis challenge models has opened several doors. For one, it is possible to map progressive immunological or haematopoietic changes during schistosome invasion in a dose‐ and time‐dependent manner. In a study, volunteers were injected with male schistosome cercariae (no eggs produced) at incremental doses (10, 20 and 30 cercariae, respectively) and followed up for up to 52 weeks [58]. All patients received at least one dose of praziquantel, or a second higher dose only when high circulatory cathodic antigen (CCA) levels persisted 3–4 weeks after the first treatment [58]. This model presented a unique advantage in mapping pre‐patency responses and cytokines and immune responses persisting after worm clearance. In a follow‐up study, Houlder et al. used single challenge models again, either with male and female cercariae and sampled peripheral blood mononuclear cells (PBMCs) up to 12 weeks post‐infection (p.i.) [35]. Serum sample analysis showed (a) higher serum IFNƔ, TNF, CXCL9, CXCL10, CXCL11 at 4 weeks, (b) IL‐10, IL‐18 and CXCL9 detected in serum at 8 weeks and (c) serum IL‐10 levels peaking at week 12 p.i. [35]. Furthermore, deep immunophenotyping of collected PBMCs revealed that severe responses in patients could be attributed to increased myeloid lineage blood cells, increased classical monocyte activation, high numbers of memory effector CD4(+) T‐cells, a decrease in various CD8(+) T‐cell populations, a decrease in CD127(+) regulatory T‐cells and increased CXCR5(+) CD38(−) memory B‐cells [35].

As elaborated earlier, severe inflammation is a potent trigger for HSPC mobilisation and can be optimally purified from peripheral blood samples [59]. There is an unexplored scope to utilise patrolling HSPCs from controlled human challenge experiments for genomic, transcriptomic and functional analyses and in characterising mild vs. severely symptomatic schistosomiasis patients. Taken together, haematopoiesis during schistosome infection is a complex premise for future investigation, with the scope of extending our findings obtained from preclinical models.

### 1.3 Immunoregulatory Schistosome Antigens as Mediators of Haematopoietic Modulation

HSPCs and the BM niche are likely to integrate worm and egg secreted antigenic load along with tissue damage and systemic and local cytokine cues. Several effector proteins identified in schistosome E/S products, adult worm proteins and SEA have regulatory functions, though there is no direct evidence proving schistosome antigens binding and regulation of HSPCs or their niche cells (Figure 2c). As an added challenge, several immunogenic schistosome antigens' cognate host cell receptors remain unknown.

In this regard, the best characterised immunomodulators are expressed by mature schistosome eggs. One of the most prominent candidates is omega‐1, an abundant SEA protein and glycosylated type 2 ribonuclease secreted by eggs. Omega‐1 glycans bind with DC‐SIGN [60], dectin‐2 [61] and mannose receptors [62]. Receptor‐mediated internalisation allows omega‐1 to utilise its ribonuclease activity in degrading ribosomal and messenger RNAs [62] and allows DCs to induce Th2 polarisation while inhibiting Th1‐ and Th17‐inducing signals [62, 63]. In this context, C‐type lectins may indirectly regulate the BM‐niche [64].

C‐type lectin receptor 2 (CLEC‐2) is expressed by both HSCs and niche‐associated megakaryocytes [65]. CLEC‐2‐deficient megakaryocytes disrupt HSC quiescence by reducing expression of thrombopoietin and CXCL12, both of which are niche‐retention signals required for HSC homing [65]. Interestingly, this study didn't assess the role of CLEC‐2 on HSCs, another unexplored mechanism of schistosome omega‐1‐dependent direct regulation of haematopoiesis.

Receptor‐mediated internalisation of schistosome antigens can allow their processing for MHCII presentation, the surface‐expressed antigen presentation protein which also helps in preserving HSC‐stemness [66]. HSCs constitutively present MHCII‐dependent antigens to restrict maladaptive transformation. On the other hand, aged HSCs can upregulate MHCII expression by activating a proinflammatory intracellular cascade, while promoting its survival and a regulatory T‐cell response in the niche [67]. Similarly, other abundant egg antigens—IPSE/1α and kappa‐5—are highly glycosylated and presumed to have similar roles to omega‐1 [68, 69], and can be tested for direct binding and modulation of HSPCs.

Haematopoietic as well as non‐haematopoietic host cells can become potential targets of pro‐fibrotic programmes elicited by SEA as well, though individual immunomodulatory candidates remain to be named and attributed with such functions. SEA is known to directly activate human dermal fibroblasts [70], and hepatic stellate cells in a TGFβ‐dependent autocrine manner [71]. TGFβ stimulates HSC quiescence, though chronic TGFβ expression can be detrimental for haematopoiesis and result in bone marrow failure [42, 44, 72]. In contrast, *S. japonicum* egg antigen p40 induces senescence [73], quiescence or induces a proinflammatory response by inducing matrix metalloprotease‐9, IL‐6 and C–C motif chemokine ligand 2 expression in hepatic stellate cells [74]. It is reasonable to suggest here that these species‐specific antigens and their orthologs and/or paralogs need to be validated individually for directly regulating HSPCs and its constituting niche cells.

Several other worm antigens, such as— *S. mansoni* cercarial invadolysin (SmCI), *S. mansoni* cathepsin B (SmCatB), asparaginyl endopeptidase (SmAE), micro‐exon gene (MEG)‐proteins paramyosin, Sm23 or MAP‐3 tetraspanin, Sm29, TPi/MAP‐4, IrV5, SmLy6B and SmTSP6, and *S. japonicum* ‐specific SjSP‐23, SjSP‐13 and SjSP‐216 are recognised immunogens [34, 75]. These worm proteins can be further characterised in regulating haematopoietic mechanisms either directly by binding HSPC/niche‐cell surface receptors, or indirectly by inactivating cytokine/chemokines essential for haematopoiesis [76].

### 1.4 Conceptual Framework for Future Studies

Schistosomes are no mere flukes silently tweaking mature immune cell populations and their effector functions [77]. Emerging evidence supports the idea that schistosomes can directly or indirectly modulate haematopoietic output in patients. Hypothesis‐driven in vivo models can be partially validated with human challenge models, when deep immunophenotypic characterisation of patrolling HSPCs is concerned (Figure 2d). However, tissue‐invasive alterations within the haematopoietic niche can only be addressed using in vivo infections, where both the central and extramedullary haematopoiesis remain as unexplored tissue responses during schistosomiasis. State‐of‐the‐art techniques such as single‐cell transcriptomic or proteomic (CODetection indEXing (CODEX)) analysis of whole BM‐samples are advantageous to study this demand‐adaptation [78]. Both techniques allow putative spatial interactions to be visualised in tissues and can be further supplemented with live‐imaging lineage‐labelled HSPCs to study their trajectory and tissue interactions in real‐time [79, 80]. In addition, temporal and spatial complexities may be evaluated by recreating this complex signalling environment ex vivo. Human bone marrow organoids have begun providing a self‐organising system that recapitulates key stromal and vascular features of the marrow niche [81, 82, 83]. These 3D‐ex vivo cultures can be seeded with primary HSPCs to test how cytokines and parasite antigens individually alter HSPCs' fitness, differentiation and niche behaviour. The scalability of such culture systems can also permit recapitulating a complex environment, while using a combination of cytokines as well as schistosome antigenic mixtures in analysing haematopoietic alterations.

Taken together, these findings support the exploration of a novel dimension of host regulatory mechanisms in schistosomiasis, in which immunohaematological processes are proposed to be central to the development of worm‐associated pathology. The scope of these research directions may extend to other helminthic infections as well, thereby opening a broad array of opportunities for advancing scientific inquiry into the aetiologies of NTDs and informing strategies for their management.

## Conflicts of Interest

The author declares no conflicts of interest.

## Data Availability

Data sharing not applicable to this article as no datasets were generated or analysed during the current study.

## References

[1] S. Warusavithana , H. Atta , M. Osman , and Y. Hutin , “Review of the Neglected Tropical Diseases Programme Implementation During 2012–2019 in the WHO‐Eastern Mediterranean Region,” PLoS Neglected Tropical Diseases 16, no. 9 (2022): e0010665. 36173943 10.1371/journal.pntd.0010665 PMC9521802

[2] Q. Li , Y. L. Li , S. Y. Guo , et al., “Global Trends of Schistosomiasis Burden From 1990 to 2021 Across 204 Countries and Territories: Findings From GBD 2021 Study,” Acta Tropica 261 (2025): 107504. 39675411 10.1016/j.actatropica.2024.107504

[3] G. Eastham , D. Fausnacht , M. H. Becker , A. Gillen , and W. Moore , “Praziquantel Resistance in Schistosomes: A Brief Report,” Frontiers in Parasitology 3 (2024): 1471451. 39817170 10.3389/fpara.2024.1471451 PMC11732111

[4] N. Vale , M. J. Gouveia , G. Rinaldi , P. J. Brindley , F. Gartner , and J. M. Correia da Costa , “Praziquantel for Schistosomiasis: Single‐Drug Metabolism Revisited, Mode of Action, and Resistance,” Antimicrobial Agents and Chemotherapy 61, no. 5 (2017): e02582–16. 28264841 10.1128/AAC.02582-16 PMC5404606

[5] M. L. Nelwan , “Schistosomiasis: Life Cycle, Diagnosis, and Control,” Current Therapeutic Research, Clinical and Experimental 91 (2019): 5–9. 31372189 10.1016/j.curtheres.2019.06.001 PMC6658823

[6] C. M. Orrico‐Ferreira , P. E. O. Cruz , J. V. B. Rios , et al., “Getting Into Host's Skin: Initial Immune Response to *Schistosoma mansoni* Infection,” Frontiers in Immunology 16 (2025): 1661465. 41479896 10.3389/fimmu.2025.1661465 PMC12753927

[7] C. D. Bourke , C. T. Prendergast , D. E. Sanin , T. E. Oulton , R. J. Hall , and A. P. Mountford , “Epidermal Keratinocytes Initiate Wound Healing and Pro‐Inflammatory Immune Responses Following Percutaneous Schistosome Infection,” International Journal for Parasitology 45, no. 4 (2015): 215–224. 25575749 10.1016/j.ijpara.2014.11.002 PMC4365920

[8] E. Hams , G. Aviello , and P. G. Fallon , “The Schistosoma Granuloma: Friend or Foe?,” Frontiers in Immunology 4 (2013): 89. 23596444 10.3389/fimmu.2013.00089 PMC3625856

[9] G. Alemu , E. Nibret , A. Munshea , et al., “Comparative Performance of Kato‐Katz, POC‐CCA and Real‐Time PCR in Detecting *Schistosoma mansoni* Infection at Different Endemicity Settings in Northwest Ethiopia: A Cross‐Sectional Study,” Tropical Medicine and Health 53, no. 1 (2025): 103. 40760670 10.1186/s41182-025-00777-7 PMC12323140

[10] M. Moon , H. W. Wu , M. Jiz , et al., “Evaluation of Sensitivity and Specificity of Kato‐Katz and Circulating Cathodic Antigen in Terms of Schistosoma Japonicum Using Latent Class Analysis,” Scientific Reports 14, no. 1 (2024): 8164. 38589377 10.1038/s41598-024-57863-9 PMC11001968

[11] M. S. Pearson , B. A. Tedla , G. G. Mekonnen , et al., “Immunomics‐Guided Discovery of Serum and Urine Antibodies for Diagnosing Urogenital Schistosomiasis: A Biomarker Identification Study,” Lancet Microbe 2, no. 11 (2021): e617–e626. 34977830 10.1016/S2666-5247(21)00150-6 PMC8683377

[12] E. J. Pearce and A. S. MacDonald , “The Immunobiology of Schistosomiasis,” Nature Reviews Immunology 2, no. 7 (2002): 499–511. 10.1038/nri843 12094224

[13] H. Lagler , C. Ay , F. Waneck , R. Gattringer , W. Graninger , and M. Ramharter , “Characterisation of Inflammatory Response, Coagulation, and Radiological Findings in Katayama Fever: A Report of Three Cases at the Medical University of Vienna, Austria,” BMC Infectious Diseases 14 (2014): 357. 24985919 10.1186/1471-2334-14-357 PMC4085376

[14] J. R. Hambrook and P. C. Hanington , “Immune Evasion Strategies of Schistosomes,” Frontiers in Immunology 11 (2020): 624178. 33613562 10.3389/fimmu.2020.624178 PMC7889519

[15] J. M. M. Angeles , V. J. P. Mercado , and P. T. Rivera , “Behind Enemy Lines: Immunomodulatory Armamentarium of the Schistosome Parasite,” Frontiers in Immunology 11 (2020): 1018. 32582161 10.3389/fimmu.2020.01018 PMC7295904

[16] S. A. Leir , O. Foot , D. Jeyaratnam , and M. B. Whyte , “Schistosomiasis and Associated Iron‐Deficiency Anaemia Presenting Decades After Immigration From Sub‐Saharan Africa,” BML Case Reports 12, no. 4 (2019): e227564. 10.1136/bcr-2018-227564 PMC6506120 31036732

[17] I. Adam , N. A. AL , O. Al‐Wutayd , and A. H. Khamis , “Prevalence of Schistosomiasis and Its Association With Anemia Among Pregnant Women: A Systematic Review and Meta‐Analysis,” Parasites & Vectors 14, no. 1 (2021): 133. 33653391 10.1186/s13071-021-04642-4 PMC7923606

[18] S. E. Butler , E. M. Muok , S. P. Montgomery , et al., “Mechanism of Anemia in *Schistosoma mansoni* ‐Infected School Children in Western Kenya,” American Journal of Tropical Medicine and Hygiene 87, no. 5 (2012): 862–867. 22987658 10.4269/ajtmh.2012.12-0248 PMC3516261

[19] J. F. Friedman , H. K. Kanzaria , and S. T. McGarvey , “Human Schistosomiasis and Anemia: The Relationship and Potential Mechanisms,” Trends in Parasitology 21, no. 8 (2005): 386–392. 15967725 10.1016/j.pt.2005.06.006

[20] E. M. Pietras , D. Reynaud , Y. A. Kang , et al., “Functionally Distinct Subsets of Lineage‐Biased Multipotent Progenitors Control Blood Production in Normal and Regenerative Conditions,” Cell Stem Cell 17, no. 1 (2015): 35–46. 26095048 10.1016/j.stem.2015.05.003 PMC4542150

[21] J. Frobel , T. Landspersky , G. Percin , et al., “The Hematopoietic Bone Marrow Niche Ecosystem,” Frontiers in Cell and Developmental Biology 9 (2021): 705410. 34368155 10.3389/fcell.2021.705410 PMC8339972

[22] M. Jagannathan‐Bogdan and L. I. Zon , “Hematopoiesis,” Development 140, no. 12 (2013): 2463–2467. 23715539 10.1242/dev.083147 PMC3666375

[23] F. Caiado , E. M. Pietras , and M. G. Manz , “Inflammation as a Regulator of Hematopoietic Stem Cell Function in Disease, Aging, and Clonal Selection,” Journal of Experimental Medicine 218, no. 7 (2021): e20201541. 34129016 10.1084/jem.20201541 PMC8210622

[24] G. A. Anderson , M. Rodriguez , and K. L. Kathrein , “Regulation of Stress‐Induced Hematopoiesis,” Current Opinion in Hematology 27, no. 4 (2020): 279–287. 32398458 10.1097/MOH.0000000000000589 PMC7328823

[25] M. H. Freedman and E. F. Saunders , “Hematopoiesis in the Human Spleen,” American Journal of Hematology 11, no. 3 (1981): 271–275. 7053225 10.1002/ajh.2830110307

[26] A. Johansson , A. Khalilnezhad , H. Takizawa , H. Mizuno , T. Suda , and T. Umemoto , “Mobilization Dynamics of Bone Marrow Hematopoietic Stem Cells During Hematopoietic Regeneration,” Experimental Hematology 138 (2024): 104281. 39009278 10.1016/j.exphem.2024.104281

[27] A. C. Tsamandas , A. B. Jain , R. B. Raikow , A. J. Demetris , M. A. Nalesnik , and P. S. Randhawa , “Extramedullary Hematopoiesis in the Allograft Liver,” Modern Pathology 8, no. 6 (1995): 671–674. 8532704

[28] S. Krishnan , K. Wemyss , I. E. Prise , et al., “Hematopoietic Stem and Progenitor Cells Are Present in Healthy Gingiva Tissue,” Journal of Experimental Medicine 218, no. 4 (2021): e20200737. 33635312 10.1084/jem.20200737 PMC7923695

[29] E. Lefrancais , G. Ortiz‐Munoz , A. Caudrillier , et al., “The Lung Is a Site of Platelet Biogenesis and a Reservoir for Haematopoietic Progenitors,” Nature 544, no. 7648 (2017): 105–109. 28329764 10.1038/nature21706 PMC5663284

[30] F. Schyrr , A. Alonso‐Calleja , A. Vijaykumar , et al., “Inducible CXCL12/CXCR4‐Dependent Extramedullary Hematopoietic Niches in the Adrenal Gland,” Blood 144, no. 9 (2024): 964–976. 38728427 10.1182/blood.2023020875

[31] C. D. Bourke , N. Nausch , N. Rujeni , et al., “Integrated Analysis of Innate, Th1, Th2, Th17, and Regulatory Cytokines Identifies Changes in Immune Polarisation Following Treatment of Human Schistosomiasis,” Journal of Infectious Diseases 208, no. 1 (2013): 159–169. 23045617 10.1093/infdis/jis524 PMC3666130

[32] B. M. Larkin , P. M. Smith , H. E. Ponichtera , M. G. Shainheit , L. I. Rutitzky , and M. J. Stadecker , “Induction and Regulation of Pathogenic Th17 Cell Responses in Schistosomiasis,” Seminars in Immunopathology 34, no. 6 (2012): 873–888. 23096253 10.1007/s00281-012-0341-9 PMC3690599

[33] R. P. De Souza , L. S. Cardoso , G. T. Lopes , et al., “Cytokine and Chemokine Profile in Individuals With Different Degrees of Periportal Fibrosis due to *Schistosoma mansoni* Infection,” Journal of Parasitology Research 2012 (2012): 394981. 23320145 10.1155/2012/394981 PMC3540765

[34] M. Egesa , L. Lubyayi , E. M. Tukahebwa , et al., “ *Schistosoma mansoni* Schistosomula Antigens Induce Th1/Pro‐Inflammatory Cytokine Responses,” Parasite Immunology 40, no. 12 (2018): e12592. 30239006 10.1111/pim.12592 PMC6492251

[35] E. L. Houlder , K. A. Stam , J. P. R. Koopman , et al., “Early Symptom‐Associated Inflammatory Responses Shift to Type 2 Responses in Controlled Human Schistosome Infection,” Science Immunology 9, no. 97 (2024): eadl1965. 38968336 10.1126/sciimmunol.adl1965

[36] T. Wijshake , J. Rose, 3rd , J. Wang , et al., “Schistosome Infection Impacts Hematopoiesis,” Journal of Immunology 212, no. 4 (2024): 607–616. 10.4049/jimmunol.2300195 PMC10872488 38169327

[37] J. H. Greenman , S. Chakraborty , L. Moss , et al., “Thrombocytopenia in Murine Schistosomiasis Is Associated With Platelet Uptake by Liver Macrophages That Have a Distinct Activation Phenotype,” PLoS Pathogens 21, no. 11 (2025): e1013732. 41296814 10.1371/journal.ppat.1013732 PMC12697973

[38] J. H. Greenman , L. Moss , S. Chakraborty , et al., “Chronic Murine Schistosomiasis Causes Aberrant Hemostasis,” Experimental Hematology 142 (2025): 104689. 39615580 10.1016/j.exphem.2024.104689

[39] N. Dietlein , X. Wang , J. Metz , et al., “Usp22 Is an Intracellular Regulator of Systemic Emergency Hematopoiesis,” Science Immunology 7, no. 78 (2022): eabq2061. 36490327 10.1126/sciimmunol.abq2061

[40] N. Sun , C. H. Lin , M. Y. Li , et al., “Clusterin Drives Myeloid Bias in Aged Hematopoietic Stem Cells by Regulating Mitochondrial Function,” Nature Aging 5, no. 8 (2025): 1510–1527. 40588652 10.1038/s43587-025-00908-z PMC12350150

[41] T. Uehata , S. Yamada , D. Ori , et al., “Regulation of Lymphoid‐Myeloid Lineage Bias Through Regnase‐1/3‐Mediated Control of Nfkbiz,” Blood 143, no. 3 (2024): 243–257. 37922454 10.1182/blood.2023020903 PMC10808253

[42] U. Blank and S. Karlsson , “TGF‐Beta Signaling in the Control of Hematopoietic Stem Cells,” Blood 125, no. 23 (2015): 3542–3550. 25833962 10.1182/blood-2014-12-618090

[43] T. Itkin , S. Gur‐Cohen , J. A. Spencer , et al., “Distinct Bone Marrow Blood Vessels Differentially Regulate Haematopoiesis,” Nature 532, no. 7599 (2016): 323–328. 27074509 10.1038/nature17624 PMC6450701

[44] J. Javier , A. Hinge , J. Bartram , J. Xu , and M. D. Filippi , “Transforming Growth Factor‐Beta Signaling Modifies the Hematopoietic Acute Inflammatory Response to Drive Bone Marrow Failure,” Haematologica 107, no. 6 (2022): 1323–1334. 34706493 10.3324/haematol.2020.273292 PMC9152956

[45] K. T. Cunningham and K. H. G. Mills , “Modulation of Haematopoiesis by Protozoal and Helminth Parasites,” Parasite Immunology 45, no. 12 (2023): e12975. 36797216 10.1111/pim.12975 PMC10909493

[46] U. M. Demel , R. Lutz , S. Sujer , et al., “A Complex Proinflammatory Cascade Mediates the Activation of HSCs Upon LPS Exposure In Vivo,” Blood Advances 6, no. 11 (2022): 3513–3528. 35413096 10.1182/bloodadvances.2021006088 PMC9198917

[47] C. Mirantes , E. Passegue , and E. M. Pietras , “Pro‐Inflammatory Cytokines: Emerging Players Regulating HSC Function in Normal and Diseased Hematopoiesis,” Experimental Cell Research 329, no. 2 (2014): 248–254. 25149680 10.1016/j.yexcr.2014.08.017 PMC4250307

[48] I. O. Farah , M. Johansson , K. Lovgren‐Bengtson , and J. Hau , “ *Schistosoma mansoni* in Mice: The Pattern of Primary Cercarial Exposure Determines Whether a Secondary Infection Post‐Chemotherapy Elicits a T Helper 1‐ or a T Helper 2‐Associated Immune Response,” Scandinavian Journal of Immunology 51, no. 3 (2000): 237–243. 10736092 10.1046/j.1365-3083.2000.00667.x

[49] M. R. Howitt , S. Lavoie , M. Michaud , et al., “Tuft Cells, Taste‐Chemosensory Cells, Orchestrate Parasite Type 2 Immunity in the Gut,” Science 351, no. 6279 (2016): 1329–1333. 26847546 10.1126/science.aaf1648 PMC5528851

[50] Y. H. Wang , P. Angkasekwinai , N. Lu , et al., “IL‐25 Augments Type 2 Immune Responses by Enhancing the Expansion and Functions of TSLP‐DC‐Activated Th2 Memory Cells,” Journal of Experimental Medicine 204, no. 8 (2007): 1837–1847. 17635955 10.1084/jem.20070406 PMC2118667

[51] N. E. Humphreys , D. Xu , M. R. Hepworth , F. Y. Liew , and R. K. Grencis , “IL‐33, A Potent Inducer of Adaptive Immunity to Intestinal Nematodes,” Journal of Immunology 180, no. 4 (2008): 2443–2449. 10.4049/jimmunol.180.4.2443 18250453

[52] R. L. Gieseck, 3rd , M. S. Wilson , and T. A. Wynn , “Type 2 Immunity in Tissue Repair and Fibrosis,” Nature Reviews Immunology 18, no. 1 (2018): 62–76. 10.1038/nri.2017.90 28853443

[53] A. Fagnan , C. Di Genua , Y. Meng , et al., “A Mechanism to Initiate Emergency Type 2 Myelopoiesis,” Nature 653, no. 8113 (2026): 212–220. 41813898 10.1038/s41586-026-10256-6 PMC13148993

[54] H. Kobayashi , C. I. Kobayashi , A. Nakamura‐Ishizu , et al., “Bacterial c‐di‐GMP Affects Hematopoietic Stem/Progenitors and Their Niches Through STING,” Cell Reports 11, no. 1 (2015): 71–84. 25843711 10.1016/j.celrep.2015.02.066

[55] A. R. de Jesus , A. Silva , L. B. Santana , et al., “Clinical and Immunologic Evaluation of 31 Patients With Acute *Schistosomiasis mansoni* ,” Journal of Infectious Diseases 185, no. 1 (2002): 98–105. 11756987 10.1086/324668

[56] T. Milner , L. Reilly , N. Nausch , et al., “Circulating Cytokine Levels and Antibody Responses to Human *Schistosoma haematobium*: IL‐5 and IL‐10 Levels Depend Upon Age and Infection Status,” Parasite Immunology 32, no. 11–12 (2010): 710–721. 21039611 10.1111/j.1365-3024.2010.01235.x PMC3033519

[57] D. N. Silva‐Teixeira , C. Contigli , J. R. Lambertucci , J. C. Serufo , and V. Rodrigues, Jr. , “Gender‐Related Cytokine Patterns in Sera of Schistosomiasis Patients With Symmers' Fibrosis,” Clinical and Diagnostic Laboratory Immunology 11, no. 3 (2004): 627–630. 15138194 10.1128/CDLI.11.3.627-630.2004 PMC404566

[58] M. C. C. Langenberg , M. A. Hoogerwerf , J. P. R. Koopman , et al., “A Controlled Human *Schistosoma mansoni* Infection Model to Advance Novel Drugs, Vaccines and Diagnostics,” Nature Medicine 26, no. 3 (2020): 326–332. 10.1038/s41591-020-0759-x 32066978

[59] N. Furer , N. Rappoport , O. Milman , et al., “A Reference Model of Circulating Hematopoietic Stem Cells Across the Lifespan With Applications to Diagnostics,” Nature Medicine 31, no. 7 (2025): 2442–2451. 10.1038/s41591-025-03716-5 PMC12283346 40579545

[60] I. van Die , S. J. van Vliet , A. K. Nyame , et al., “The Dendritic Cell‐Specific C‐Type Lectin DC‐SIGN Is a Receptor for *Schistosoma mansoni* Egg Antigens and Recognizes the Glycan Antigen Lewis x,” Glycobiology 13, no. 6 (2003): 471–478. 12626400 10.1093/glycob/cwg052

[61] M. Ritter , O. Gross , S. Kays , et al., “ *Schistosoma mansoni* Triggers Dectin‐2, Which Activates the Nlrp3 Inflammasome and Alters Adaptive Immune Responses,” Proceedings of the National Academy of Sciences of the United States of America 107, no. 47 (2010): 20459–20464. 21059925 10.1073/pnas.1010337107 PMC2996650

[62] B. Everts , L. Hussaarts , N. N. Driessen , et al., “Schistosome‐Derived Omega‐1 Drives Th2 Polarization by Suppressing Protein Synthesis Following Internalization by the Mannose Receptor,” Journal of Experimental Medicine 209, no. 10 (2012): 1753–1767. 22966004 10.1084/jem.20111381 PMC3457738

[63] E. van Liempt , S. J. van Vliet , A. Engering , et al., “ *Schistosoma mansoni* Soluble Egg Antigens Are Internalized by Human Dendritic Cells Through Multiple C‐Type Lectins and Suppress TLR‐Induced Dendritic Cell Activation,” Molecular Immunology 44, no. 10 (2007): 2605–2615. 17241663 10.1016/j.molimm.2006.12.012

[64] A. Nakamura‐Ishizu , K. Takubo , H. Kobayashi , K. Suzuki‐Inoue , and T. Suda , “CLEC‐2 in Megakaryocytes Is Critical for Maintenance of Hematopoietic Stem Cells in the Bone Marrow,” Journal of Experimental Medicine 212, no. 12 (2015): 2133–2146. 26552707 10.1084/jem.20150057 PMC4647260

[65] P. Hernandez‐Malmierca , D. Vonficht , A. Schnell , et al., “Antigen Presentation Safeguards the Integrity of the Hematopoietic Stem Cell Pool,” Cell Stem Cell 29, no. 5 (2022): 760–75.e10. 35523139 10.1016/j.stem.2022.04.007 PMC9202612

[66] J. Li , M. J. Williams , H. J. Park , et al., “STAT1 Is Essential for HSC Function and Maintains MHCIIhi Stem Cells That Resist Myeloablation and Neoplastic Expansion,” Blood 140, no. 14 (2022): 1592–1606. 35767701 10.1182/blood.2021014009 PMC7614316

[67] W. Liao , C. Liu , K. Yang , et al., “Aged Hematopoietic Stem Cells Entrap Regulatory T Cells to Create a Prosurvival Microenvironment,” Cellular & Molecular Immunology 20, no. 10 (2023): 1216–1231. 37644165 10.1038/s41423-023-01072-3 PMC10541885

[68] L. F. Pennington , A. Alouffi , E. C. Mbanefo , et al., “H‐IPSE Is a Pathogen‐Secreted Host Nucleus‐Infiltrating Protein (Infiltrin) Expressed Exclusively by the *Schistosoma haematobium* Egg Stage,” Infection and Immunity 85, no. 12 (2017): e00301–17. 28923894 10.1128/IAI.00301-17 PMC5695104

[69] P. Kalantari , S. C. Bunnell , and M. J. Stadecker , “The C‐Type Lectin Receptor‐Driven, Th17 Cell‐Mediated Severe Pathology in Schistosomiasis: Not All Immune Responses to Helminth Parasites Are Th2 Dominated,” Frontiers in Immunology 10 (2019): 26. 30761125 10.3389/fimmu.2019.00026 PMC6363701

[70] D. J. Wyler and J. W. Tracy , “Direct and Indirect Effects of Soluble Extracts of *Schistosoma mansoni* Eggs on Fibroblast Proliferation In Vitro,” Infection and Immunity 38, no. 1 (1982): 103–108. 6890530 10.1128/iai.38.1.103-108.1982 PMC347703

[71] X. Qi , Y. Pu , F. Chen , et al., “Schistosome Egg Antigen Stimulates the Secretion of miR‐33‐Carrying Extracellular Vesicles From Macrophages to Promote Hepatic Stellate Cell Activation and Liver Fibrosis in Schistosomiasis,” PLoS Neglected Tropical Diseases 17, no. 5 (2023): e0011385. 37253066 10.1371/journal.pntd.0011385 PMC10256196

[72] L. Jiang , X. Han , J. Wang , et al., “SHP‐1 Regulates Hematopoietic Stem Cell Quiescence by Coordinating TGF‐Beta Signaling,” Journal of Experimental Medicine 215, no. 5 (2018): 1337–1347. 29669741 10.1084/jem.20171477 PMC5940262

[73] J. Chen , T. Xu , D. Zhu , et al., “Egg Antigen p40 of Schistosoma Japonicum Promotes Senescence in Activated Hepatic Stellate Cells by Activation of the STAT3/p53/p21 Pathway,” Cell Death & Disease 7, no. 7 (2016): e2315. 27468691 10.1038/cddis.2016.228 PMC4973363

[74] B. J. Anthony , K. R. James , G. N. Gobert , G. A. Ramm , and D. P. McManus , “ *Schistosoma japonicum* Eggs Induce a Proinflammatory, Anti‐Fibrogenic Phenotype in Hepatic Stellate Cells,” PLoS One 8, no. 6 (2013): e68479. 23840855 10.1371/journal.pone.0068479 PMC3686876

[75] A. Floudas , C. D. Cluxton , J. Fahel , et al., “Composition of the *Schistosoma mansoni* Worm Secretome: Identification of Immune Modulatory Cyclophilin A,” PLoS Neglected Tropical Diseases 11, no. 10 (2017): e0006012. 29073139 10.1371/journal.pntd.0006012 PMC5681295

[76] J. R. Hambrook and P. C. Hanington , “A Cercarial Invadolysin Interferes With the Host Immune Response and Facilitates Infection Establishment of *Schistosoma mansoni* ,” PLoS Pathogens 19, no. 2 (2023): e1010884. 36730464 10.1371/journal.ppat.1010884 PMC9928134

[77] G. Schramm , A. Suwandi , A. Galeev , et al., “Schistosome Eggs Impair Protective Th1/Th17 Immune Responses Against Salmonella Infection,” Frontiers in Immunology 9 (2018): 2614. 30487793 10.3389/fimmu.2018.02614 PMC6246638

[78] L. Li , I. Rottmann , B. R. Saeed , et al., “High‐Dimensional Spatiotemporal Single‐Cell Atlas and 3D Imaging of the Bone Marrow Microenvironment During CML Progression,” Blood 147, no. 22 (2026): 2648–2665. 41592325 10.1182/blood.2025029824 PMC13277661

[79] M. L. R. Haltalli , S. Watcham , N. K. Wilson , et al., “Manipulating Niche Composition Limits Damage to Haematopoietic Stem Cells During Plasmodium Infection,” Nature Cell Biology 22, no. 12 (2020): 1399–1410. 33230302 10.1038/s41556-020-00601-w PMC7611033

[80] M. K. Scott , O. Akinduro , and C. Lo Celso , “In Vivo 4‐Dimensional Tracking of Hematopoietic Stem and Progenitor Cells in Adult Mouse Calvarial Bone Marrow,” Journal of Visualized Experiments 91 (2014): e51683. 10.3791/51683 PMC4828047 25225854

[81] D. Janagama and S. K. Hui , “3‐D Cell Culture Systems in Bone Marrow Tissue and Organoid Engineering, and BM Phantoms as In Vitro Models of Hematological Cancer Therapeutics—A Review,” Materials 13, no. 24 (2020): 5609. 33316977 10.3390/ma13245609 PMC7763362

[82] K. Ren , E. Li , I. Aydemir , et al., “Development of iPSC‐Derived Human Bone Marrow Organoid for Autonomous Hematopoiesis and Patient‐Derived HSPC Engraftment,” Blood Advances 9, no. 1 (2025): 54–65. 39471483 10.1182/bloodadvances.2024013361 PMC11732577

[83] Y. Shen , C. Benlabiod , E. Watson , et al., “comBO: A Combined Human Bone and Lympho‐Myeloid Bone Marrow Organoid for Preclinical Modeling of Hematopoietic Disorders,” Cell Stem Cell 33, no. 3 (2026): 421–37.e7. 41734765 10.1016/j.stem.2026.01.010 PMC7618947

